# Clinicopathological and prognostic significance of platelet-to-lymphocyte ratio in patients with hepatocellular carcinoma

**DOI:** 10.18632/oncotarget.13244

**Published:** 2016-11-09

**Authors:** Wei Song, Kai Wang, Fu-ping Zhong, You-wen Fan, Lang Peng, Shu-bing Zou

**Affiliations:** ^1^ Department of Hepatobiliary Surgery, The Second Affiliated Hospital of Nanchang University, Nanchang, China

**Keywords:** platelet to lymphocyte ratio (PLR), hepatocellular carcinoma, biomarker, prognosis, meta-analysis

## Abstract

The platelet-to-lymphocyte ratio (PLR) is reported to be a prognostic factor in multiple malignancies. The aim of this study was to assess its prognostic value in hepatocellular carcinoma (HCC). We performed comprehensive searches of electronic databases for relevant studies. A total of eleven studies comprising 2,507 patients were included. Elevated PLR was significantly associated with poor overall survival (OS) (HR = 1.78; 95% CI = 1.36-2.34; P < 0.001) and disease-free survival (DFS)/recurrence-free survival (RFS) (HR = 1.82; 95% CI = 1.56-2.13; P < 0.001). The findings from most subgroup analyses were consistent with those from the overall analysis. In addition, a high PLR correlated with tumor size > 3 cm, TNM stage, lymph node metastasis, distant metastasis, and vascular invasion. We therefore conclude that elevated pretreatment PLR may be predicative of a poor prognosis in patients with HCC.

## INTRODUCTION

Hepatocellular carcinoma (HCC) is the second leading cause of cancer-related death worldwide, with an estimated 500,000 to 1 million deaths per year [1, 2]. More than two-thirds of patients are diagnosed at an advanced stage, when curative treatments, including hepatic resection, radio-frequency ablation, and liver transplantation, are no longer an option [3]. Despite advances in surgical techniques and perioperative management, the overall prognosis of HCC remains poor due to a high recurrence rate and intrahepatic metastasis after curative resection [4]. It is therefore vital to identify novel predictive biomarkers that can be used to improve prognosis and select appropriate therapeutic strategies.

Systemic inflammatory responses play a critical role in the pathogenesis and progression of cancer [5]. Inflammation indicators, such as serum ferritin (SF), neutrophil-to-lymphocyte ratio (NLR), lymphocyte-to-monocyte ratio (LMR), platelet-to-lymphocyte ratio (PLR), and C-reactive protein (CRP) have been identified as prognostic indicators in various cancers [6–10]. Studies have also shown that platelets play multiple roles during inflammatory response processes. High platelet counts can promote cancer progression by facilitating neoangiogenesis, production of adhesion molecules and increases in early metastatic niches [11, 12]. By contrast, lymphocytes hinder malignant progression through tumoral infiltration by multiple lymphocyte subtypes. Low lymphocyte counts are often seen in patients with advanced cancer [13]. Moreover, a high platelet-to-lymphocyte ratio (PLR), which is defined as absolute platelet counts divided by lymphocyte counts, is reportedly linked to an unfavorable prognosis in multiple malignancies [10, 14, 15].

Nevertheless, the prognostic value of PLR in HCC has not yet been fully elucidated. Furthermore, there has been no systematic review or meta-analysis to determine the reliability and degree of its prognostic value. We therefore conducted a meta-analysis to assess the effects of pretreatment PLR on OS and DFS/RFS as well as the associations between PLR and the clinicopathological features of patients with HCC.

## RESULTS

### Study characteristics

**Figure 1:**
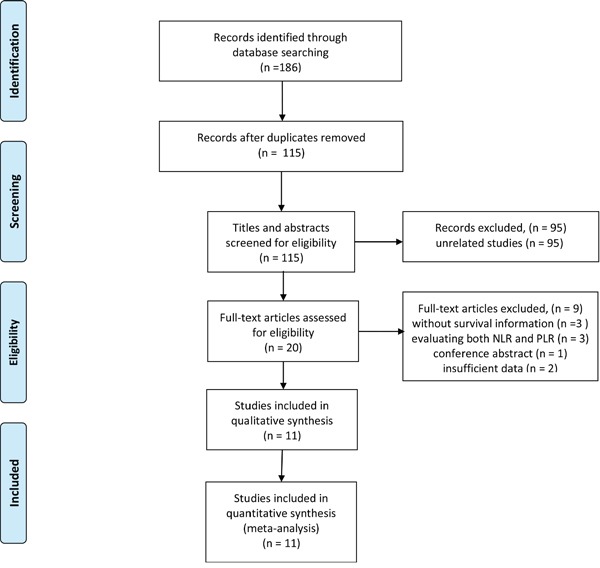
Flow diagram of the study selection process

The literature search of electronic databases identified a total of 186 articles. After duplicates removal, 115 articles were screened for eligibility. Of these, 95 were excluded through titles and abstracts, leaving 20 articles for detailed evaluation. Nine studies did not meet the inclusion criteria and were therefore excluded. Ultimately, 11 eligible studies, comprising a total of 2,507 patients, were considered eligible for the meta-analysis [16–26]. The PRISMA flow diagram of the study selection process was shown in Figure 1.

Of 11 studies, 10 studies were published in 2015 or later. Eight studies were from China, one from UK, one from USA, and one from Singapore. The sample sizes ranged from 116 to 414. Ten studies investigated the prognostic role of PLR in OS, and 5 studies explored the prognostic impact of PLR in DFS/RFS. The cut-off values for PLR ranged from 87.87 to 290, 4 studies used a PLR cut-off value ≥ 150, while 7 studies used a PLR < 150. HRs and 95% CIs were extracted directly from the 9 studies. HRs in 2 studies were estimated by Kaplan-Meier survival curves. Characteristics of included studies are shown in Table 1.

**Table 1:** Characteristics of the studies included in the meta-analysis

Author	Year	Country	Age (years)	Study type	Gender (M/F)	Ethnicity	Follow-up (months)	Treatment	No. of patients	Stage	Cut-off value	Survival analysis	HR estimate	Analysis
D'Emic	2016	USA	60(28-85)	R	52/64	Caucasian	12(5.3-18.1)	Mixed	116	Mixed	290	OS/PFS	Reported	MV/UV
Fan	2015	China	49(23-75)	R	87/45	Asian	11(4–46)	Chemotherapy	132	Non- metastatic	137	OS	Reported	MV/UV
Goh	2016	Singapore	66 (21-85)	R	142/24	Asian	23(0-170)	Surgery	166	Mixed	290	OS/RFS	Reported	UV
Ji	2016	China	51(21-79)	R	285/36	Asian	NA	Surgery	321	Mixed	115	OS	Reported	MV/UV
Li(1)	2015	China	57 (19-86)	R	211/32	Asian	2.7(0.1-44.8)	Mixed	243	Metastatic	111.23	OS	Reported	MV/UV
Li(2)	2015	China	59.5±12.1	R	329/85	Asian	NA	Mixed	414	Non- metastatic	87.87	RFS	Estimated	MV
Neofytou	2014	UK	NA	R	52/88	Caucasian	33(1-103)	Mixed	140	Metastatic	150	OS/DFS	Reported	MV/UV
Peng	2015	China	50(21-78)	R	191/28	Asian	36.4(3-85.9)	Surgery	219	Mixed	ΔPLR 2.875	OS/RFS	Reported	MV/UV
Tian	2016	China	56 (26-77)	R	107/15	Asian	22(3-118)	Chemotherapy	122	Mixed	96.13	OS	Reported	MV/UV
Xia	2015	China	49.4(19-71)	R	308/35	Asian	33.7(9.5-132)	Mixed	343	Mixed	125	OS/DFS	Estimated	MV
Xue	2015	China	53.05±11.48	R	258/33	Asian	9	Chemotherapy	291	Mixed	150	OS	Reported	MV/UV

R: retrospective; H: high expression; L: low expression; OS: overall survival; DFS: disease-free survival; RFS: recurrence-free survival; MV: multivariate; NA: not available.

### Quality assessment

In methodological quality of studies, the global quality score ranged 50.0% to 70.0%, with a median of 67.6% (Table 2). The subscore of laboratory methodology had the lowest value, with a median quality score of 5.8 out of 14. The most poorly described items were the blinding evaluation, tissue sample conservation, and description of the revelation test procedure.

**Table 2:** Methodological assessments of the studies included in the meta-analysis

Author	Global score (%)	Scientific design (/10)	Laboratory methodology (/14)	Generalizability (/12)	Results analysis (/8)
D'Emic	65.9	7	6	9	7
Fan	72.7	7	6	12	7
Goh	65.9	8	6	8	7
Ji	70.5	9	6	10	6
Li(1)	70.5	8	4	11	8
Li(2)	61.4	7	6	10	4
Neofytou	70.5	9	6	8	8
Peng	68.2	7	6	10	7
Tian	72.7	8	6	10	8
Xia	50.0	6	6	6	4
Xue	75.0	9	6	10	8

### Meta-analysis

#### Overall survival

**Figure 2:**
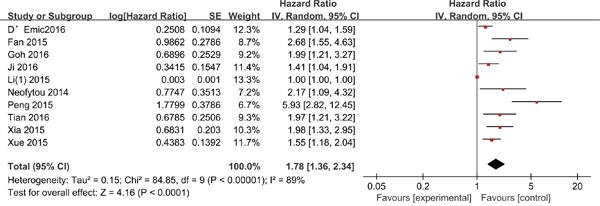
Forest plots for the association between PLR expression and OS

Ten studies involving 2,093 patients investigated the association between PLR and OS. Elevated PLR was significantly associated with poor OS (HR = 1.78; 95% CI = 1.36-2.34; P < 0.001; Figure 2). The test for heterogeneity was significant, thus, the random-effects model was used (I^2^ = 89%; P < 0.001). To detect the potential heterogeneity, subgroup analyses stratified by ethnicity, treatment, sample size, disease stage, HR estimation, analysis method, ELCWP score, and the cut-off value of PLR (Table 3). Exploratory subgroup analysis according to ethnicity showed that elevated PLR had more significantly prognostic value for OS in Asian populations (HR = 1.88; 95% CI = 1.33-2.65; P < 0.001). Pooled HRs for OS stratified by treatment, the negative effect of elevated PLR on OS was observed in patients receiving chemotherapy (HR = 1.77; 95% CI = 1.43-2.21; P < 0.001), surgery (HR = 2.38; 95% CI = 1.17-4.84; P = 0.02), and mixed methods (HR = 1.40; 95% CI = 1.01-1.92; P = 0.04). In the subgroup analysis by disease stage, patients with high PLR had significantly worse OS in patients with non-metastatic disease (pooled HR = 2.68; 95% CI = 1.55-4.63; P < 0.001) and mixed subgroup (HR = 1.79; 95% CI = 1.39-2.29; P < 0.001). The cut-off values ranged from 87.87 to 290. We stratified cut-off values into two subgroups: <150 and ≥150. Stratification by the cut-off value showed the OS rate was significantly worse in all subgroups. In addition, subgroup analyses suggested that high PLR predicted poor OS in patient with HCC, regardless of the sample size (<200 and ≥200), HR estimation (reported and estimated), analysis method (univariate and multivariate), and ELCWP score (<70 and ≥70).

**Table 3:** Pooled hazard ratios (HRs) for OS according to subgroup analyses

Subgroup	No. of studies	No. of patients	Effects model	HR (95% CI)	P value	Heterogeneity	
						I^2^ (%)	P_h_
Overall	10	2093	Random	1.78 (1.36, 2.34)	<0.001	89	< 0.001
Ethnicity							
Asian	8	1837	Random	1.88 (1.33, 2.65)	<0.001	91	<0.001
Caucasian	2	256	Random	1.50 (0.94, 2.40)	0.09	51	0.15
Treatment							
Chemotherapy	3	545	Fixed	1.77 (1.43, 2.21)	<0.001	40	0.19
Surgery	3	706	Random	2.38 (1.17, 4.84)	0.02	84	0.002
Mixed	6	842	Random	1.40 (1.01, 1.92)	0.04	86	< 0.001
Sample size							
<200	5	676	Random	1.85 (1.35, 2.53)	<0.001	58	0.05
≥200	5	1417	Random	1.71 (1.14, 2.57)	0.009	92	< 0.001
Disease stage							
Non-metastatic	1	132	-	2.68 (1.55, 4.63)	<0.001	-	-
Mixed (non-metastatic & metastatic)	7	1578	Random	1.79 (1.39, 2.29)	<0.001	69	0.004
Metastatic	2	383	Random	1.36 (0.65, 2.85)	0.41	79	0.03
Cut-off for PLR							
<150	6	1380	Random	1.96 (1.25, 3.06)	0.003	91	< 0.001
≥150	4	713	Fixed	1.46 (1.25, 1.71)	<0.001	31	0.23
HR estimation							
Reported	9	1750	Random	1.76 (1.32, 2.33)	<0.001	89	< 0.001
Estimated	1	343	-	1.98 (1.33, 2.95)	<0.001	-	-
Analysis method							
Univariate	1	166	-	1.99 (1.21, 3.27)	0.006	-	-
Multivariate	9	1927	Random	1.76 (1.33, 2.34)	<0.001	90	<0.001
ELCWP score							
<70	4	844	Random	2.14 (1.29, 3.56)	0.003	83	< 0.001
≥70	6	1249	Random	1.61 (1.15, 2.24)	0.005	87	< 0.001

### Disease-free survival/recurrence-free survival

**Figure 3:**
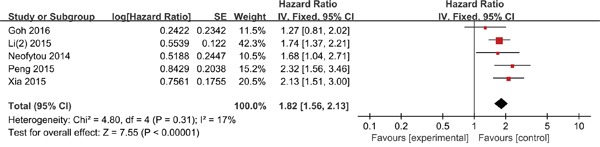
Forest plots for the association between PLR expression and DFS/RFS

Five studies comprising 1,282 patients evaluated the association between PLR and DFS/RFS. In comparison with a low PLR, a high PLR was significantly correlated with worse DFS/RFS (HR = 1.82; 95% CI = 1.56-2.13; P < 0.001; Figure 3), without significant heterogeneity (I^2^ = 17%; P = 0.31).

### Clinicopathological parameters

In the meta-analysis, we identified 12 clinical factors to explore the impact of PLR on the clinical features in HCC. Pooled data revealed that a high PLR was significantly related to tumor size (> 3 cm vs. < 3 cm; HR = 1.67, 95% CI: 1.11-2.52, P = 0.01), TNM stage (III-IV vs. I-II; HR = 2.20, 95% CI: 1.11-4.33, P = 0.02), lymph node metastasis (pos vs. neg; HR = 1.62, 95% CI: 1.01-2.60, P = 0.04), distant metastasis (pos vs. neg; HR = 2.38, 95% CI: 1.23-4.60, P = 0.01), and vascular invasion (pos vs. neg; HR = 1.70, 95% CI: 1.20-2.43, P = 0.003). Whereas no significant association was found with gender (male vs. female), cirrhosis (yes vs. no), AFP (>400 ng/mL vs. <400 ng/mL), Child-Pugh classification (B/C vs. A), differentiation (low vs. moderate/high), tumor number (> 3 cm vs. < 3 cm), tumor size (> 5 cm vs. < 5 cm), and tumor distribution (bilobar vs. unilobar). The correlation between PLR expression and clinicopathological parameters of HCC is shown in Table 4.

**Table 4:** Meta-analysis of the association between PLR and clinicopathological features of HCC

Characteristics	No. of studies	No. of patients	OR (95% CI)	p	Heterogeneity	
					I^2^ (%)	P_h_
Gender (male vs. female)	9	2070	0.79 (0.61, 1.01)	0.06	0	0.57
Cirrhosis (yes vs. no)	4	1090	1.88 (0.22, 16.26)	0.57	97	< 0.001
AFP(>400 ng/mL vs. <400 ng/mL)	4	764	1.13 (0.83, 1.52)	0.44	34	0.21
Child-Pugh classification (B/C vs. A)	4	911	0.94 (0.62, 1.42)	0.76	46	0.14
Differentiation ( low vs. moderate/high)	3	976	1.01 (0.42, 2.42)	0.99	72	0.03
Tumor number (> 3 cm vs. < 3 cm)	3	615	0.45 (0.14, 1.47)	0.19	87	< 0.001
Tumor size (> 3 cm vs. < 3 cm)	3	473	1.67 (1.11, 2.52)	0.01	26	0.26
Tumor size (> 5 cm vs. < 5 cm)	2	483	2.07 (0.29, 14.62)	0.46	93	< 0.001
Tumor distribution (bilobar vs. unilobar)	2	306	1.17 (0.68, 2.03)	0.56	26	0.24
TNM stage (III-IV vs. I-II)	3	531	2.20 (1.11, 4.33)	0.02	61	0.07
Lymph node metastasis (pos vs. neg)	2	365	1.62 (1.01, 2.60)	0.04	0	0.83
Distant metastasis (pos vs. neg)	1	243	2.38 (1.23, 4.60)	0.01	-	-
Vascular invasion (pos vs. neg)	4	860	1.70 (1.20, 2.43)	0.003	0	0.41

### Sensitivity analysis and publication bias

Each single study was removed each time to estimate the influence of individual data sets on the combined HR. The results of sensitivity analysis showed that no study had a significant effect on the observed effect size (pooled HR), indicating the robustness of our findings. Evidence of publication bias was observed for OS (P = 0.074 for Begg's test and P < 0.001 for Egger's test), while no significant publication bias was detected for DFS/RFS by both the Begg's test (P = 1.000) and the Egger's test (P = 0.864). Using “trim and fill” method, we assessed the impact of this bias and the HR for OS didn't show a shift.

## DISCUSSION

HCC has been shown to be an inflammation-induced cancer [27]. Approximately 80% of HCC cases are related to chronic HBV or HCV infections [28]. Recently, several clinical studies showed that a high PLR correlates with worse prognosis and clinicopathologic features in patients with HCC [18, 19, 29]. Moreover, Tian et al. found that elevated pretreatment PLR is predictive of poor OS among patients with HBV-related HCC [24]. Similarly, Fan et al. reported that a high PLR correlates significantly with a poor prognosis and metastasis in recurrent HCC patients [17]. PLR has also been shown to correlate with recurrence and survival rates in patients with HCC [29]. These findings suggest PLR could serve as a promising prognostic or therapeutic target for HCC patients. To our knowledge, this is the first meta-analysis investigating the correlation between PLR and survival or clinicopathological features in patients with HCC.

We identified 11 studies involving 2,507 patients that evaluated the clinical relevance and prognostic value of PLR in patients with HCC. This meta-analysis showed that elevated PLR is an unfavorable prognostic factor for OS and DFS/RFS in patients with HCC. Subgroup analyses revealed that the negative prognostic effect of elevated PLR remained substantial despite different sample sizes, cut-off values, treatment methods, HR estimation methods, analysis methods, and ELCWP scores. However, a stratified analysis showed that a high PLR had no prognostic efficiency for OS in Caucasian or metastatic patients. Additionally, when we further analyzed the correlations between pretreatment PLR and clinicopathologic parameters, we found that elevated PLR was linked with tumor size > 3 cm, TNM stage, lymph node metastasis, distant metastasis, and vascular invasion.

The mechanisms responsible for the association between high PLR and poor outcome in HCC remain unclear. However, mounting evidence suggests that systemic inflammation plays an important role in tumor initiation and progression by contributing to genomic instability, genetic mutations, cancer cell proliferation, angiogenesis, and hematogenous metastasis [13, 30]. Cancer-related inflammation can suppress antitumor immunity by recruiting immunosuppressive cells such as myeloid-derived suppressor cells and regulatory T cells, resulting in tumor progression [31, 32].

It has been suggested that there is cross-talk between the inflammatory response and tumor progression [5, 13, 33]. It is now generally accepted that platelets bind VEGF, PDGF, FGF, and TGF-β family proteins, enabling platelets to act as a reservoir for secreted growth factors that increase tumor angiogenesis, cell proliferation, migration, and metastasis [34–36]. Tumor-infiltrating lymphocytes (TILs) are important immune cells found within tumors and are responsible for antitumor immune responses [37]. High numbers of TILs correlate with favorable clinical outcomes [38, 39]. In HCC patients, high levels of tumor-infiltrating CD4+ T lymphocytes are associated with a lower recurrence rate and better prognosis [40]. This suggests PLR combined with the effects of platelets and lymphocytes may be predictive of prognosis in patients with HCC.

This meta-analysis has several limitations. First, significant heterogeneity was found among studies. However, subgroup analyses showed that the heterogeneity diminished or disappeared in patients receiving chemotherapy, and the cut-off for PLR ≥150. Moreover, the stability of our results was confirmed by sensitivity analysis. Second, the cut-off value for PLR differed among the studies. This may be a significant contributor to the heterogeneity. Third, publication bias was detected for OS. As we know, studies with negative results are less likely to be published than those with positive results. Additionally, only published articles were included, and they were all written in English. Therefore, the summary statistics obtained may not approximate the actual average. However, using the “Trim and Fill” method to evaluate this bias, the pooled effect size remained significant. This indicates the reliability of our results. Fourth, HRs and their 95% CIs were extracted from univariable analyses in only one study and estimated from Kaplan-Meier survival curves in two studies. Thus, the prognostic value of PLR may be overestimated.

In conclusion, our meta-analysis confirms that an elevated pretreatment PLR is significantly associated with poor survival in conjunction with advanced tumor stage and positive metastasis in HCC patients. This suggests pretreatment PLR could provide essential information that informs prognosis and treatment decisions for patients HCC.

## MATERIALS AND METHODS

### Search strategies

A comprehensive search of MEDLINE, EMBASE, and Cochrane databases from the inception to July 2016 was performed. The following MeSH terms and text words were used in combination: “hepatocellular carcinoma” or “HCC” or “liver cancer” or “liver tumor” or “liver neoplasms” or “liver cell carcinoma”, “platelet to lymphocyte ratio” or “PLR” or “platelet lymphocyte ratio” or “platelet-lymphocyte ratio”, “prognostic” or “prognosis” or “survival” or “recurrence” or “outcome”. In addition, the references of eligible studies, pertinent reviews, and meta-analyses in this field were screened.

### Study selection

The criteria for inclusion were listed as follows: (1) the diagnosis of HCC was pathologically confirmed. (2) assessing the prognostic value of pretreatment PLR on OS, DFS and/or RFS; (3) reporting a sufficient information to estimate the hazard ratio (HR) and 95% confidence interval (CI); (4) reporting a dichotomous cut-off value for PLR; and (5) original high-quality English articles. The exclusion criteria were: (1) abstract, reviews, conference, case reports, and expert opinion; (2) reporting PLR only as a continuous variable; (3) lacking essential information for calculating an HR and 95% CI; and (4) overlapping or duplicate data.

### Data extraction

The following information was captured using data abstraction forms:

first author's name, year of publication, study design, country, ethnicity, patient ages and genders, number of patients, disease stage, treatment, follow-up, cut-off value, outcome measures (HRs for OS, DFS, or RFS, as well as their 95% CIs), survival analysis methods, and clinicopathological features. HRs were directly extracted from multivariate or univariate analyses or estimated from Kaplan-Meier survival curves independently by two reviewers and any discrepancies were resolved a third reviewer.

### Quality assessment

The quality of each study was evaluated in accordance with the revised ELCWP scoring scale described by Steel et al. [41]. Each item was assessed using an ordinal scale (possible values: 2, 1, 0). The overall score include the following four categories: (1) scientific design: 0-10; (2) laboratory methodology: 0-14; (3) generalizability: 0-12; (4) results analysis: 0-8. The total scores ranged from 0 to 44. The final scores are expressed as percentages, with a higher scores reflecting a better methodological quality.

### Statistical analyses

The meta-analysis was conducted by RevMan 5.3 software (Cochrane Collaboration, Copenhagen, Denmark) and STATA 12.0 (College Station, TX, USA). HRs and their 95% CIs were searched in the original articles or extrapolated using methods described by Tierney and Parmar [42, 43]. The associations between PLR and clinicopathologic features were expressed as odds ratios (ORs) and its 95 % CIs. Statistical heterogeneity among eligible studies was estimated using Cochrane's Q statistic and I^2^ statistic [44]. A p-value < 0.1 for the Q-test or I^2^ >50% was considered statistically significant. When there was no statistically significant heterogeneity, we used the fixed-effects model for pooling the results; otherwise, the random-effects model was applied. Subgroup analyses stratified by ethnicity, treatment, sample size, disease stage, HR estimation, analysis method, ELCWP score, and the cut-off value of PLR. In order to evaluate the robustness of conclusions, sensitivity analysis was performed by excluding single study at a time from the meta-analysis to explore its influence on the pooled HR for OS. Publication bias was evaluated using Begg's funnel plots and Egger's tests. When publication bias was identified, we used the “Trim and Fill” method to re-estimate a corrected effect size after adjustment for publication bias [45]. P < 0.05 was defined as statistically significant.
